# Supplementary material for: Humanized COVID‐19 decoy antibody effectively blocks viral entry and prevents SARS‐CoV‐2 infection
Source: EMBO Mol Med. 2020 Nov 30;13(1):e12828. doi: 10.15252/emmm.202012828 (PMC7799362; doi:10.15252/emmm.202012828)
Supplement: Supplementary file 1 — Appendix [file EMMM-13-e12828-s001.pdf]

Appendix Table S1

List of the exact P-values for main and EV figures

Figure	Panel	Comparaison	P value	Dose response
Figure 2	A	buffer vs 0.64 µg/mL ACE2-Fc	0.00048	
		buffer vs 1.25 µg/mL ACE2-Fc	0.00103	
		buffer vs 2.5 µg/mL ACE2-Fc	0.00010	
		buffer vs 5 µg/mL ACE2-Fc	0.00010	
	E	50 µg/mL IgG vs 50 µg/mL ACE2-Fc	0.02744	
	F	50 µg/mL IgG vs 50 µg/mL ACE2-Fc	0.02490	
Figure 3	D (left)	(293T/ACE2) 100 µg/mL IgG vs 25 µg/mL ACE2-Fc	0.00199	
		(293T/ACE2) 100 µg/mL IgG vs 100 µg/mL ACE2-Fc	< 0.00001	
		(H1975/ACE2) 100 µg/mL IgG vs 25 µg/mL ACE2-Fc	0.00011	
		(H1975/ACE2) 100 µg/mL IgG vs 100 µg/mL ACE2-Fc	0.00002	
		(H1975/ACE2) 25 µg/mL vs 100 µg/mL ACE2-Fc	0.02281	+
	D (right)	(293T/ACE2) 100 µg/mL IgG vs 25 µg/mL ACE2-Fc	0.00371	
		(293T/ACE2) 100 µg/mL IgG vs 100 µg/mL ACE2-Fc	< 0.00001	
		(H1975/ACE2) 100 µg/mL IgG vs 25 µg/mL ACE2-Fc	0.00817	
		(H1975/ACE2) 100 µg/mL IgG vs 100 µg/mL ACE2-Fc	0.00001	
		(H1975/ACE2) 25 µg/mL vs 100 µg/mL ACE2-Fc	0.01009	+
Figure 4	A	(293T/ACE2) 100 µg/mL IgG vs 25 µg/mL ACE2-Fc	0.03492	
		(293T/ACE2) 100 µg/mL IgG vs 50 µg/mL ACE2-Fc	0.00749	
		(293T/ACE2) 100 µg/mL IgG vs 100 µg/mL ACE2-Fc	0.00301	
		(H1975/ACE2) 100 µg/mL IgG vs 25 µg/mL ACE2-Fc	0.02698	
		(H1975/ACE2) 100 µg/mL IgG vs 50 µg/mL ACE2-Fc	0.00227	
		(H1975/ACE2) 100 µg/mL IgG vs 100 µg/mL ACE2-Fc	0.00144	
		(293T/ACE2) 12.5 µg/mL vs 50 µg/mL ACE2-Fc	0.00552	+
		(293T/ACE2) 12.5 µg/mL vs 100 µg/mL ACE2-Fc	0.00966	+
		(293T/ACE2) 25 µg/mL vs 50 µg/mL ACE2-Fc	0.04592	+
		(293T/ACE2) 25 µg/mL vs 100 µg/mL ACE2-Fc	0.00477	+
		(293T/ACE2) 50 µg/mL vs 100 µg/mL ACE2-Fc	0.01345	+
		(H1975/ACE2) 12.5 µg/mL vs 50 µg/mL ACE2-Fc	0.00249	+
		(H1975/ACE2) 12.5 µg/mL vs 100 µg/mL ACE2-Fc	0.00111	+
		(H1975/ACE2) 25 µg/mL vs 50 µg/mL ACE2-Fc	0.02319	+
		(H1975/ACE2) 25 µg/mL vs 100 µg/mL ACE2-Fc	0.00928	+
		(H1975/ACE2) 50 µg/mL vs 100 µg/mL ACE2-Fc	0.04645	+
	D	Control vs 100 µg/mL ACE2-Fc	0.00017	
		Control vs 300 µg/mL ACE2-Fc	0.00013	
Figure 5	A	(w/o trypsin) 100 µg/mL IgG vs 100 µg/mL ACE2-Fc	0.02034	
		(w/o trypsin) 100 µg/mL IgG vs 200 µg/mL ACE2-Fc	0.01803	
		(with trypsin) 200 µg/mL IgG vs 200 µg/mL ACE2-Fc	0.01739	
		(w/o trypsin) 6.25 µg/mL vs 100 µg/mL ACE2-Fc	0.03824	+
		(w/o trypsin) 6.25 µg/mL vs 200 µg/mL ACE2-Fc	0.03591	+
		(w/o trypsin) 12.5 µg/mL vs 100 µg/mL ACE2-Fc	0.00914	+
		(w/o trypsin) 12.5 µg/mL vs 200 µg/mL ACE2-Fc	0.00681	+
		(w/o trypsin) 50 µg/mL vs 100 µg/mL ACE2-Fc	0.04631	+
		(w/o trypsin) 50 µg/mL vs 200 µg/mL ACE2-Fc	0.03604	+
	B	100 µg/mL IgG vs 100 µg/mL ACE2-Fc	0.02888	
		100 µg/mL IgG vs 200 µg/mL ACE2-Fc	0.00918	
		6.25 µg/mL ACE2-Fc vs 50 µg/mL ACE2-Fc	0.02908	+

		6.25 μg/mL ACE2-Fc vs 100 μg/mL ACE2-Fc	0.01567	+
		6.25 μg/mL ACE2-Fc vs 200 μg/mL ACE2-Fc	0.00648	+
		12.5 μg/mL ACE2-Fc vs 100 μg/mL ACE2-Fc	0.02174	+
		12.5 μg/mL ACE2-Fc vs 200 μg/mL ACE2-Fc	0.00779	+
		25 μg/mL ACE2-Fc vs 200 μg/mL ACE2-Fc	0.01284	+
		50 μg/mL ACE2-Fc vs 200 μg/mL ACE2-Fc	0.01983	+
	F	400 μg/mL IgG vs 200 μg/mL ACE2-Fc	0.00217	
		400 μg/mL IgG vs 400 μg/mL ACE2-Fc	0.00519	
Figure 6	B	NTU3 IgG vs ACE2-Fc	0.00198	
		NTU13 IgG vs ACE2-Fc	0.00794	
		NTU14 IgG vs ACE2-Fc	0.00967	
		NTU25 IgG vs ACE2-Fc	0.00216	
		NTU27 IgG vs ACE2-Fc	0.00473	
	C	NTU14 IgG vs ACE2-Fc	0.00184	
		NTU25 IgG vs ACE2-Fc	0.00014	
		NTU27 IgG vs ACE2-Fc	0.00035	
Figure 7	B	buffer vs 20 μg/mL ACE2-Fc	0.01640	
		buffer vs 40 μg/mL ACE2-Fc	0.01600	
		buffer vs 60 μg/mL ACE2-Fc	0.01282	
	C	(CD107a) 40 μg/mL ACE2 vs 40 μg/mL ACE2-Fc	0.02719	
		(IFN γ) 40 μg/mL ACE2 vs 40 μg/mL ACE2-Fc	0.00009	
		(TNF- α) 40 μg/mL ACE2 vs 40 μg/mL ACE2-Fc	0.02603	
Figure EV4		(293T/ACE2) 100 μg/mL IgG vs 25 μg/mL ACE2-Fc	0.00133	
		(293T/ACE2) 100 μg/mL IgG vs 50 μg/mL ACE2-Fc	0.00058	
		(293T/ACE2) 100 μg/mL IgG vs 100 μg/mL ACE2-Fc	0.00029	
		(H1975/ACE2) 100 μg/mL IgG vs 25 μg/mL ACE2-Fc	0.03371	
		(H1975/ACE2) 100 μg/mL IgG vs 50 μg/mL ACE2-Fc	0.01211	
		(H1975/ACE2) 100 μg/mL IgG vs 100 μg/mL ACE2-Fc	0.00723	
