# Supplementary material for: Humanized COVID‐19 decoy antibody effectively blocks viral entry and prevents SARS‐CoV‐2 infection
Source: EMBO Mol Med. 2020 Nov 30;13(1):e12828. doi: 10.15252/emmm.202012828 (PMC7799362; doi:10.15252/emmm.202012828)
Supplement: Supplementary file 2 — Expanded View Figures PDF [file EMMM-13-e12828-s002.pdf]

Expanded View Figures

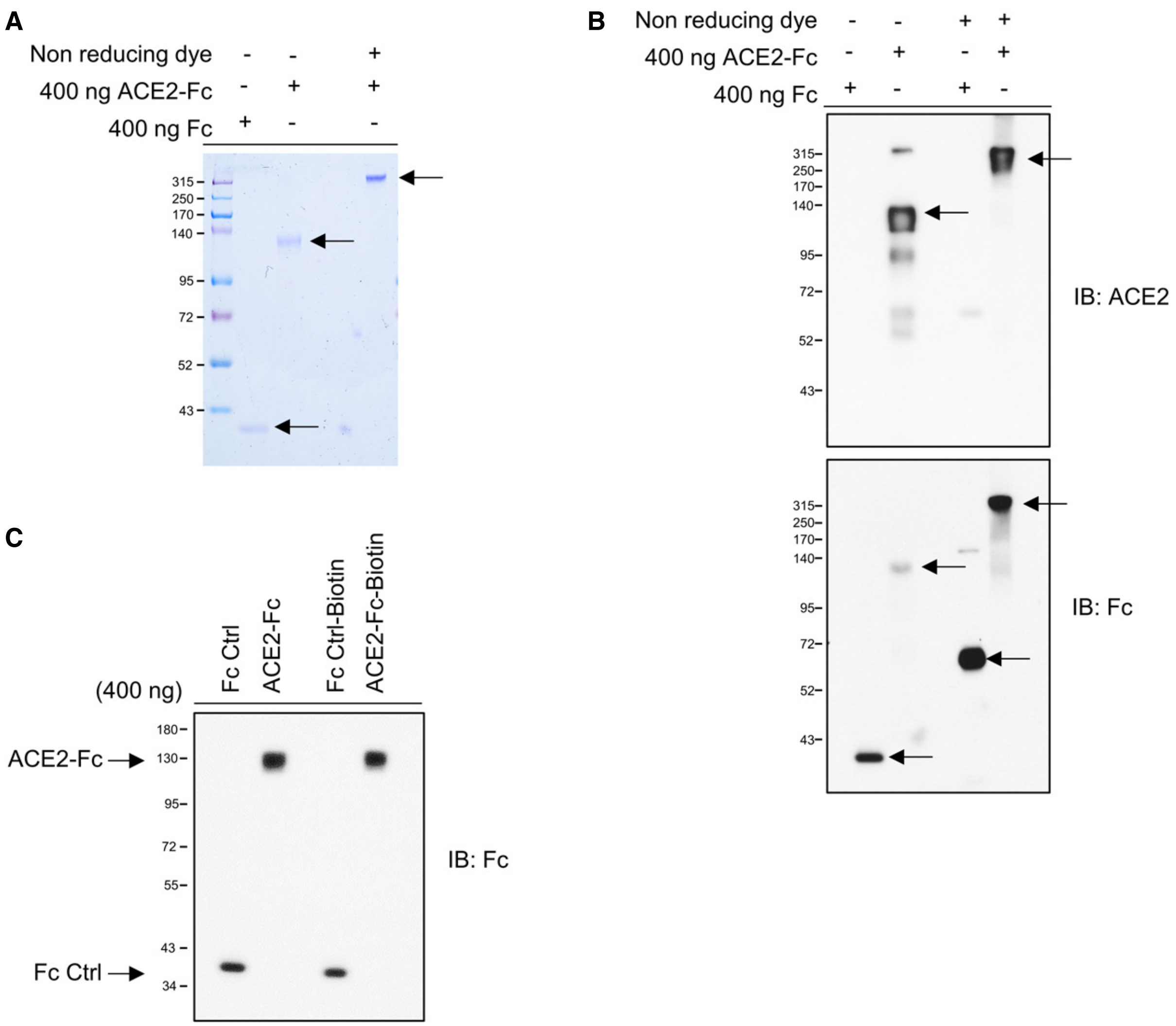

Figure EV1. Coomassie Brilliant Blue staining and immunoblotted sodium dodecyl sulfate-polyacrylamide gel electrophoresis (SDS–PAGE) for ACE2-Fc.

A The purity and molecular size of purified ACE2-Fc. The ACE2-Fc in the supernatants were purified by Protein G Sepharose (Merck). A single band of ACE2-Fc was observed by Coomassie Brilliant Blue staining using reducing or nonreducing loading dye. The black arrows indicate the location of the induced target proteins.

B Recognition of the decoy antibody by anti-ACE2 antibody and anti-human IgG Fc antibody. Purified ACE2-Fc formed homodimers in nonreducing SDS–PAGE. The black arrows indicate the location of the induced target proteins.

C The purity of biotin-labeled ACE2-Fc or Fc control was immunoblotted with an anti-human IgG Fc antibody. IB: immunoblotted with the indicated antibodies.

 © 2021 The Authors

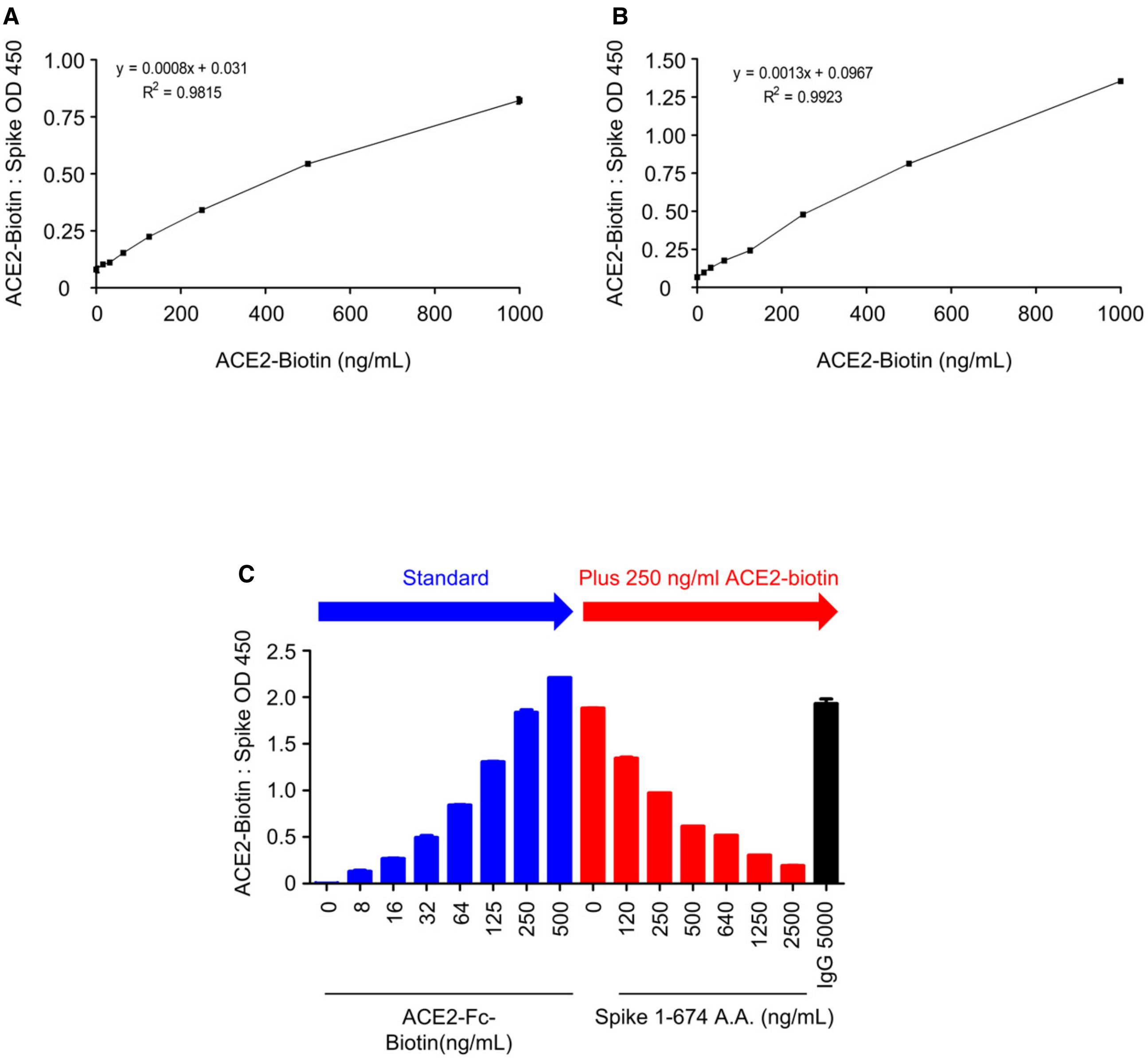

Figure EV2. ELISA binding curve of ACE2-Fc to the Spike protein.

A, B ACE2-Fc was serially diluted and dispensed into wells coated with Spike 1–674 (S1) (A) or Spike 319–591 (RBD-SD1) (B), and the binding activity was analyzed by ELISA.

C The soluble form of Spike 1–674 protein blocked the ACE2-Biotin: Spike 1–674 interaction. RBD: receptor-binding domain; SD: connector domain. Error bars represent the standard deviation (SD), $n = 2$.

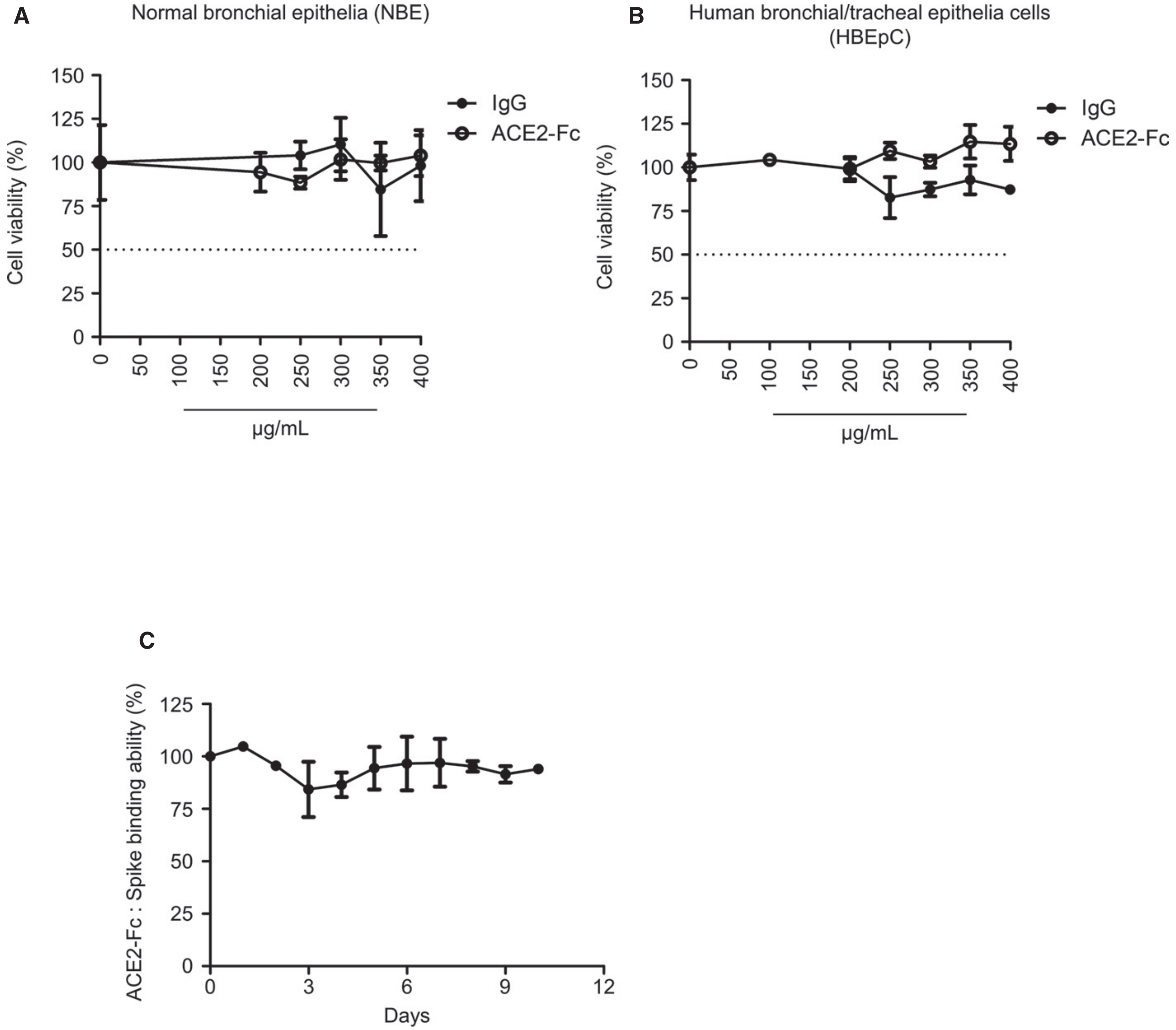

Figure EV3. *In vitro* cytotoxicity and plasma stability and of ACE2-Fc.

A, B Two normal human bronchial epithelial cells were incubated with ACE2-Fc and normal human IgG at the indicated concentrations for 72 h, and cell viability was analyzed by MTS assay. Error bars represent the standard deviation (SD), $n = 2$ in left; $n = 3$ in right. The dotted line represents the 50% of cell viability.

C *In vitro* serum stability of ACE2-Fc. ACE2-Fc was incubated with 50% normal human serum at 37°C for up to 10 days. At the indicated time points, samples were collected to quantify the binding ability of ACE2-Fc to Spike proteins by ELISA. Error bars represent the standard deviation (SD), $n = 3$. Experiments were performed at least three times with similar results.

 © 2021 The Authors

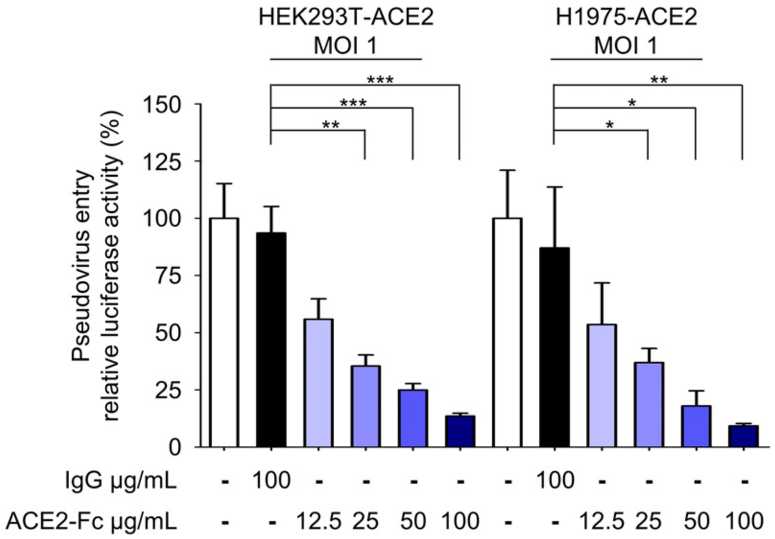

Figure EV4. Inhibition of pseudovirus entry by ACE2-Fc at higher virus input.

The same experimental protocol was conducted as in Fig 4A, except with a higher virus input. ACE2-Fc blocked Spike-expressing pseudotyped lentivirus entry into HEK293T-ACE2 and H1975-ACE2 cells. MOI (multiplicity of infection) = 1. Virus entry was determined by measuring luciferase activity. R.I. U: (R.I. U = relative infection unit. Error bars represent the standard deviation (SD), $n = 3$. Statistical analysis was performed by unpaired two-tail t-test. *$P < 0.05$, **$P < 0.01$, ***$P < 0.001$.

© 2021 The Authors

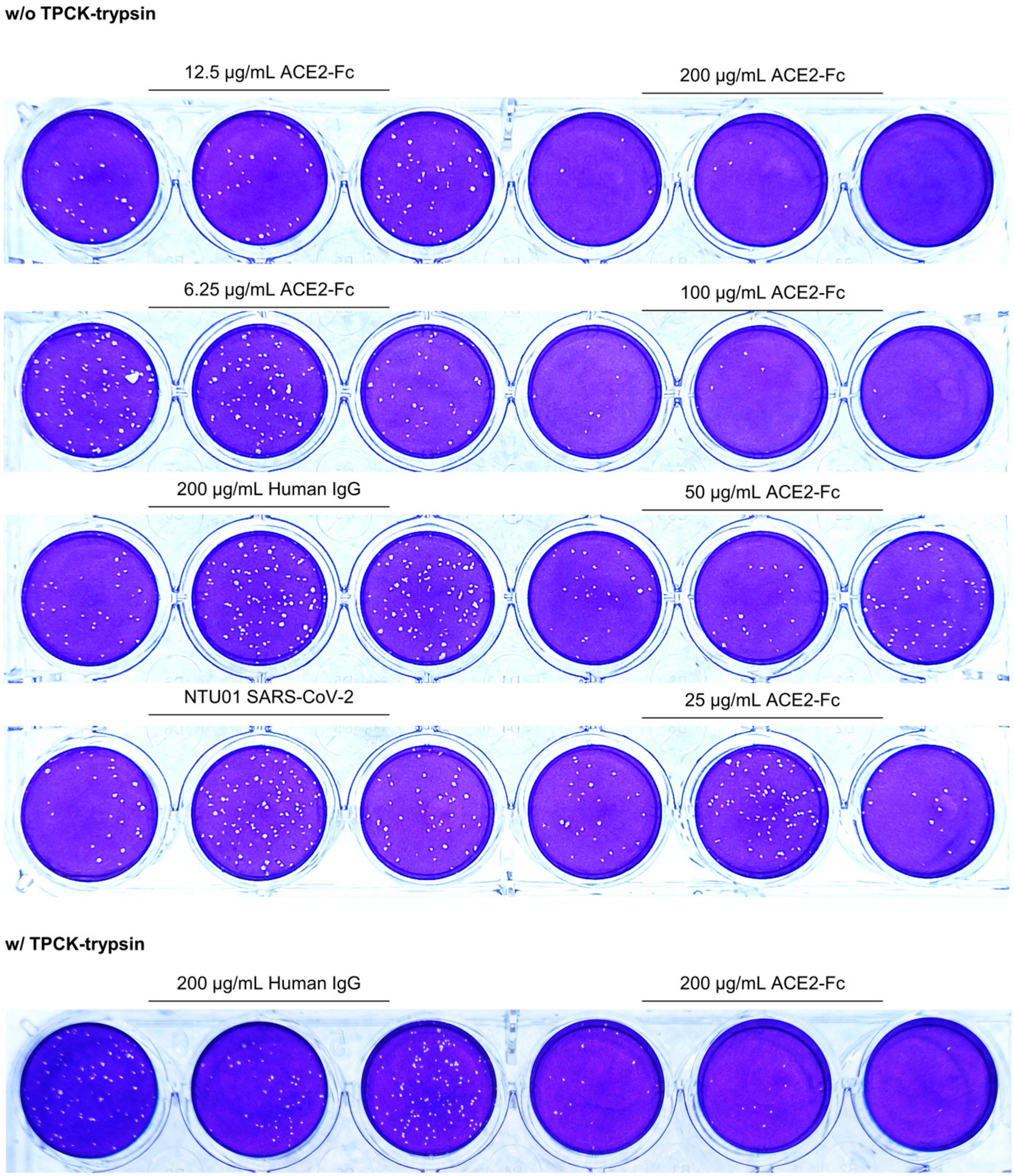

Figure EV5. Plaque reduction assay.

SARS-CoV-2 (4000 plaque-forming units, PFUs) was incubated with antibodies at the indicated amounts for 1 h at 37°C before adding to the Vero E6 cell monolayer for another hour. After the mixtures were removed, the cells were washed and replaced with overlay medium for 5 days. Plaque formation was determined by crystal violet staining. NTU01: SARA-CoV-2 that was isolated from a female patient suffering from COVID-19 infection at National Taiwan University Hospital.

 © 2021 The Authors
